# Effects of *Scrophularia oxysepala* Methanolic Extract on Early Stages of Dimethylhydrazine-Induced Colon Carcinoma in Rats: Apoptosis Pathway Approach

**DOI:** 10.34172/apb.2022.085

**Published:** 2021-09-29

**Authors:** Ali Namvaran, Mehdi Fazeli, Safar Farajnia, Gholamreza Hamidian, Hassan Rezazadeh

**Affiliations:** ^1^Department of Pharmacology and Toxicology, School of Veterinary Medicine, Shiraz University, Shiraz, Iran.; ^2^Drug Applied Research Center, Tabriz University of Medical Sciences, Tabriz, Iran.; ^3^Department of Pharmacology and Toxicology, Faculty of Pharmacy, Tabriz University of Medical Sciences, Tabriz, Iran.; ^4^Department of Basic Science, Faculty of Veterinary Medicine, University of Tabriz, Tabriz, Iran.

**Keywords:** Scrophularia oxysepala, Colon cancer, 1, 2-dimethylhydrazine, Apoptosis, Caspase 3

## Abstract

***Purpose:*** Colorectal cancer is one of the most prevalent cancers, worldwide. The present study aimed to examine the effects of *Scrophularia oxysepala* (SO) methanolic extract on 1,2-dimethylhydrazine (DMH) induced colon cancer model in the Wistar rats.

***Methods:*** The animals administered DMH (40 mg/kg/S.C.) biweekly for 2 weeks to induce aberrant crypt foci (ACF). Other groups of animals were given the SO extract (50, 100 and 200 mg/kg/orally once/day) either before or after the DMH treatments. In the end, all animals were killed and at necropsy, the colon samples examined. The ACF, aberrant crypt (AC), crypt multiplicity (CM), caspase 3 protein and apoptosis measurement were performed.

***Results:*** The SO extract significantly (*P*<0.001) decreased the number of AC, ACF, and CM in all pre- and post-treated groups and caused significant increases in caspase 3 and apoptosis as compared to the DMH group. However, post-treated animals showed significantly more effective than pre-treatment groups. Methanolic extract of SO showed a chemopreventive potential, by effectively reducing the number of AC, ACF, and CM and increasing caspase 3 protein and apoptosis.

***Conclusion:*** One of the possible mechanisms might be involved in the induction of apoptosis through the caspase 3 mediated pathway.

## Introduction

In developing countries and economically developed countries, cancer incidence and mortality rates are increasing because of aging and population growth. Lung, breast, colorectal, liver, stomach and cervical cancers are the most common type.^1^ It has been estimated that 589, 430 cancer death and 1 658 370 new cases of cancer occurred during 2015 in the USA, however, Colorectal cancer is more common in both genders and a large number of cases were diagnosed during the year of 2014, also enormous death due to colorectal cancer among men and women was reported in the USA.^2,3^ Thus, research to find an effective way to treat different kinds of cancers, especially colorectal cancer, is a priority.

Using natural and folklore medicine to treat cancer is much in common among different cultures, because of plenty of anti-cancer components in various herbs. Researches about health effects of plant-derived extracts and phytochemicals are going to be popular through past decades and some of these products used in a variety of modern drugs.^4-6^ This popularity leads to produce more than 60% of all drugs like vinblastine, etoposide, paclitaxel, vincristine, camptothecin derivatives, which are obtained from native resources.^7,8^ *Scrophulariaceae* family are angiosperms plants distributed in Asia/North America, containing a variety of species and genera.^9^ Therapeutic effects of *Scrophularia* species in inflammatory diseases, as anti-oxidant, bactericidal, wound healing, and against psoriasis have been reported.^10-13^ Effects* of S*. *oxysepala* on some kinds of cancer cell lines were studied in previous researches.^4,7,14^ However, its impact on gastrointestinal cancers has not been reported. Therefore, studying the mechanism of anticancer drugs is very important. The existing anticancer therapies are concentrated on producing apoptosis as a major key procedure in cell development. In the course of embryonic differentiation and growth, apoptosis is a key event. After the embryonic stage, it is essential in regulating homeostasis. In apoptosis, proteolytic enzymes called caspases play a key role in the cascade. The execution pathway begins by caspase 3 is the terminal step of both intrinsic and extrinsic apoptosis pathways, leads to motivation and completion by cell death.

There are several experimental models to induce colon cancer. One of the most frequently used chemical agents to induced colon cancer in animal models is 1,2-dimethylhydrazine (DMH).^15,16^ One of the widely used models of experimental colon carcinogenesis is the DMH model. It contributes high similarities to colon cancer in humans, including similarities in response to preventive and promotional agents.^15,17^ Nowadays, it is a broadly used model for investigating chemopreventive agents, environmental and dietary conditions in the carcinogenesis of large intestine. Also, it is used to determine different molecular and morphological mechanisms involved in the development of different stages of colon cancer to clarify new treatment. ^17^

This study aimed to examine the effectiveness of pre-treatment and treatment of *Scrophularia oxysepala*(SO) extract on early stages of colon carcinoma induced by DMH in rats and possible apoptosis progress.

## Materials and Methods

###  Animals

 Male Wistar rats (8 weeks old) were procured from Tabriz University of Medical Sciences. The rats maintained in standard conditions, including; 4 animals per polypropylene cages covered with metallic grids in a room with controlled temperature and humidity (22 ± 2ºC, 55 ± 10%, respectively) with a 12-hour light-dark cycle. For 2 weeks accommodation period, a standard commercial rodent diet was used for feeding with water ad libitum access. Experiments were carried out in accordance with the guide for the Care and Use of Laboratory Animals (National Institutes of Health Publication No 85-23, revised 1985). Also the protocol was approved by the Committee on Animal Research of Tabriz University of Medical Sciences. All efforts were made to minimize the number of animals that were used and their suffering degree.

###  Experimental design

 Rats were allocated into eight groups randomly, 8 animals in each group. The first group received normal saline and a standard rodent diet. The second group was given four subcutaneous (S.C.) injection of DMH (40 mg/kg b.w.) twice a week, for 2 weeks. Groups 3-5 given four S.C. injections of DMH (40 mg/kg b.w.) twice a week, for 2 weeks and then received SO extract orally for 4 weeks, 50, 100 and 200 mg/kg, respectively (post-treatment groups). Groups 5-8 received SO extract orally for 4 weeks, 50, 100, and 200 mg/kg/daily, respectively, prior given four S.C. injection of DMH (40 mg/kg b.w.) twice a week (pre-treatment groups). Groups that received only EDTA solution (DMH vehicle, 37 mg/100 mL distilled water) omitted in the result analysis because of no effects on the results.

###  Tissue and histology processing

The time of sampling was at the end of 6 weeks from beginning, all animals were killed and the colon was removed at necropsy, then, represented colon samples were fixed by 10% phosphate-buffered formalin solution for 48 hours. The incidences of the multiplicity of murine aberrant crypt foci (ACF), adenomas, and adenocarcinomas are higher in the middle and distal colon than in the proximal colon of animals treated with DMH.^18-20^ So, the main focus of our analysis preformed on the distal and middle portions of the colon.

The number of ACF per cm^2^ and the number of aberrant crypts (ACs) in each focus were determined by light microscopic examination at 40x magnification as was described by previous researchers.^21,22^ ACF was determined by the surrounding crypts by their slit-like opening, darker staining, increased size, an elliptical shape, and pericryptal zone. Crypt multiplicity (CM) was also defined as the number of ACs in each microscopic focus. The neoplasm was classified according to the histopathological classification proposed by Sunter et al.^23^ All the scores were set on by one observer that was blinded to treatment groups during the scoring of crypts and checked at random by a second study.

Also, a semi-quantitative histological examination performed on samples according to the following grades.^24,25^

 Grade 0: Normal structure.

 Grade 1: Sloughing of surface epithelium, mild mucosal damage.

 Grade 2: Loss of one-third of mucosal crypts, moderate damage.

 Grade 3: Loss of two-thirds of mucosal crypts, extensive damage.

 Grade 4: Mural infarct, mucosal and submucosal necrosis was present.

 Grade 5: Transmural infarct, necrosis in areas throughout the thickness of the intestinal wall.

***Scrophularia*** ***oxysepala*** **extraction**

 The main parts of (aerial parts) SO were gathered from Kalibar mountain areas in North-West of Iran (Eastern Azerbaijan province) during May-June 2018. The identity was verified by the specimen of Dr. Abbas Delazar (Pharm D., Ph. D of Pharmacognosy) held in the School of Pharmacy, Tabriz University of Medical Sciences using morphologically compared with the herbarium.

 The powdered SOaerial parts were dried and used for extraction. 20 g of prepared SO powder was mixed with 200 mL of 80% methyl alcohol. Forty-eight hours after the first step, the mixture was leached, and the solvent was extracted in a rotary evaporator adjusted at 60ºC to medium speed. The fluid was dried at 50ºC, and the obtained powder was used in the present study.

###  TUNEL assay

 Apoptosis detection was performed using the terminal dUTP nick end-labeling (TUNEL) method. In the TUNEL method, enzymatically labeled the endonuclease-generated DNA breaks by terminal transferase with biotin-conjugated UTP, which is possible to be detected by an immunoperoxidase reaction. The process was executed on deparaffinized tissue sections according to the protocol of In Situ Cell Death Detection Kit POD (Roche Diagnostics GmbH, Germany) as per manufacturer’s instruction. Fifty crypt columns (vertically oriented colonic crypts) were used to quantitatively evaluate apoptotic cells by counting the TUNEL positive cells among those cells under light microscopy (1000×). TUNEL-positive cells per 100 cells were considered as the apoptotic index.

###  Caspase 3 evaluation

 Ab151283 Pro Caspase 3 ELISA (Enzyme-Linked Immunosorbent Assay) kit was used to assay perform of caspase 3 protein in tissue lysates. All procedures followed by the instruction of ELISA kit producer. Shortly, colon lysates were prepared by homogenization of the tissue, which was first minced and thoroughly rinsed in PBS to remove feces and blood. The homogenate was then suspended to 10mg/ml in PBS. The homogenate was solubilized by combining with an equal volume of 2x extraction buffer of a kit containing 1 mM phenylmethylsulfonyl fluoride and incubated on ice for 20 minutes. Centrifugation was performed at 18000× g for 20 minutes at 4ºC. The supernatants were transferred into clean tubes and the pellets were discarded. Regarding instruction, assay was performed immediately for samples.

###  Statistical analysis

All the data represented in this study are expressed as mean ± SEM (standard error of the mean). One-way analysis of variance (ANOVA) followed by Tukey’s post hoc test, Kruskal–Wallis and then Mann–Whitney U tests for Histological examination was performed to determine the significant differences between groups. The level of statistical significance was set at *P* < 0.05.

## Results and Discussion

 In the present study, the chemopreventive potential of SOon methanolic extract was investigated in colon cancer. ACF induced by DMH was used as substitute biomarkers of colon cancer. Our experiment assessed antiproliferative and chemopreventive potentials based on the number, multiplicity of the abnormal crypt, apoptosis, caspase 3 and histological evaluations. All animals were survived until the end of the experiment procedure.

###  ACF, AC and CM development

**Figure 1:**
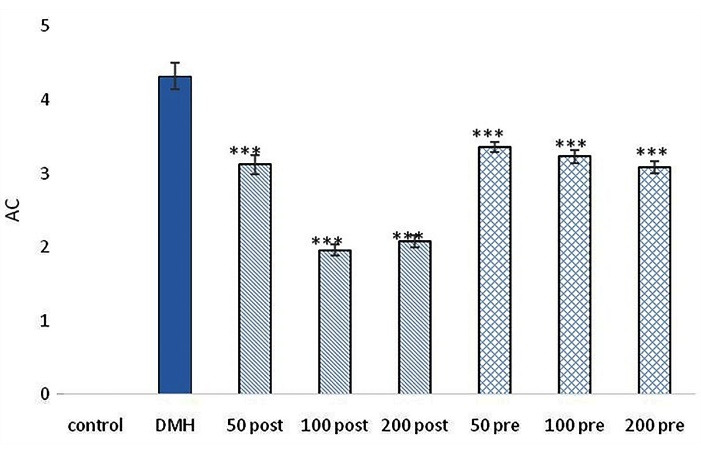
Chemo-preventive effects of *Scrophularia oxysepala* (SO)hydro-methanol extract on the pre-treatment and post-treatment number of aberrant crypt (AC) per cm length in the distal colon of DMH (1,2-dimethylhydrazine) induced colon carcinogenesis. Animals received 50 mg/kg, 100 mg/kg and 200 mg/kg of extract after cancer induction called post-treatment groups (50 post, 100 post, and 200 post respectively) and animals which received 50 mg/kg, 100 mg/kg and 200 mg/kg of the extract before cancer induction called pre-treatment groups (50 pre, 100 pre, and 200 pre respectively).. Data are presented as means (n = 8) ± SEM. *** represent significant difference (*P* < 0.001) relative to DMH group

**Figure 2:**
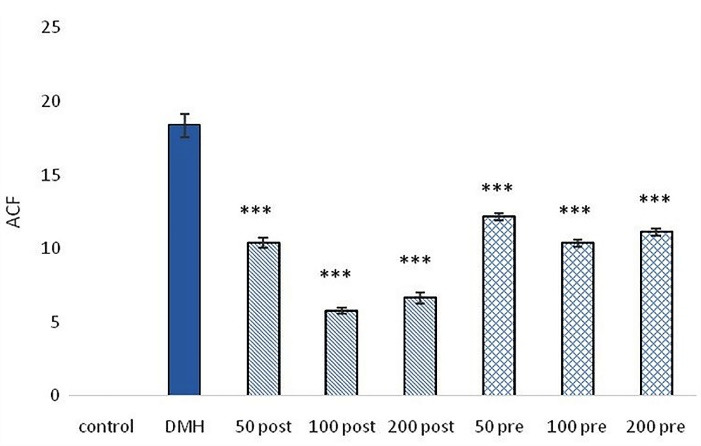
Chemo-preventive effects of *Scrophularia oxysepala* (SO)hydro-methanol extract on the pre-treatment and post-treatment number of aberrant crypt foci (ACF) per cm length in the distal colon of DMH (1,2-dimethylhydrazine) treatment induced colon carcinogenesis. Animals received 50 mg/kg, 100 mg/kg and 200 mg/kg of extract after cancer induction called post-treatment groups (50 post, 100 post, and 200 post respectively) and animals which received 50 mg/kg, 100 mg/kg and 200 mg/kg of the extract before cancer induction called pre-treatment groups (50 pre, 100 pre, and 200 pre respectively). Data are presented as means (n = 8) ± SEM. *** represent significant difference (*P* < 0.001) relative to DMH group

**Figure 3:**
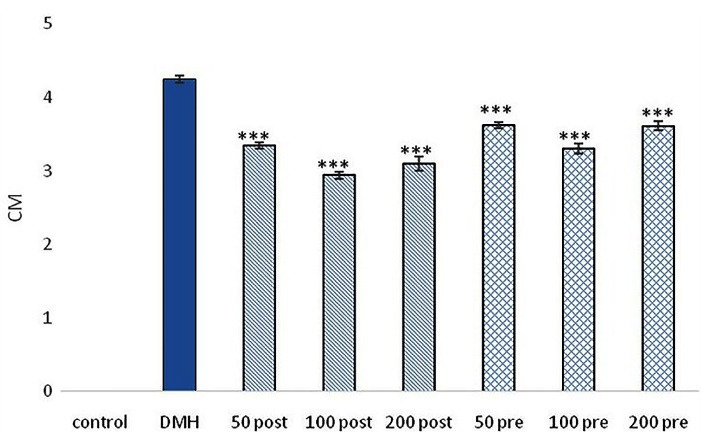
Chemo-preventive effects of *Scrophularia oxysepala* (SO)hydro-methanol extract on the pre-treatment and post-treatment number of crypt multiplicity (CM) per cm length in the distal colon of DMH (1,2-dimethylhydrazine) treatment induced colon carcinogenesis. Animals received 50 mg/kg, 100 mg/kg and 200 mg/kg of extract after cancer induction called post-treatment groups (50 post, 100 post, and 200 post respectively) and animals which received 50 mg/kg, 100 mg/kg and 200 mg/kg of the extract before cancer induction called pre-treatment groups (50 pre, 100 pre, and 200 pre respectively). Data are presented as means (n = 8) ± SEM. *** represent significant difference (*P* < 0.001) relative to DMH groups

Developed the pre-neoplastic lesions were observed in all animals which received the carcinogen. The control group didn’t show any ACs, ACF and CM. Only in the DMH group, the mean ± SEM of ACs, ACF and CM was 4.32 ± 0.18, 18.36 ± 0.79 and 4.25 ± 0.05, respectively. All intervention treatment groups which received pre-treatment and post-treatment of SO extract showed a significant reduction in the mean ± SEM of ACs, ACF and CM compared with DMH group (*P* < 0.001). The least number of AC, ACF, and CM was observed in the post-treatment group treated with 100 mg/kg. Post-treatment groups showed significant (*P* < 0.01) response in reducing ACs, ACF, and CM compared with pre-treatment groups (Figures 1, 2 and 3).

###  Apoptosis

**Figure 4:**
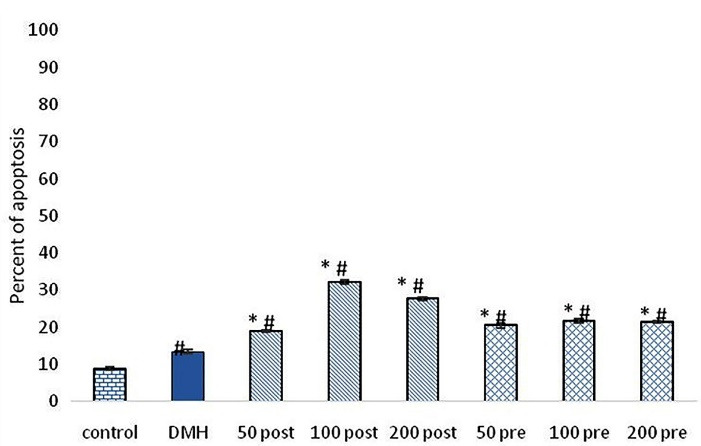
The apoptotic effects of *Scrophularia oxysepala* (SO)methanolic extract, detected by TUNEL assay. Animals received 50 mg/kg, 100 mg/kg and 200 mg/kg of extract after cancer induction called post-treatment groups (50 post, 100 post, and 200 post respectively) and animals which received 50 mg/kg, 100 mg/kg and 200 mg/kg of the extract before cancer induction called pre-treatment groups (50 pre, 100 pre, and 200 pre respectively).” Data are presented as means (n = 8) ± SEM. * represent significant difference (*P* < 0.001) relative to DMH group. # represent significant difference (*P* < 0.001) relative to control group.

**Figure 5:**
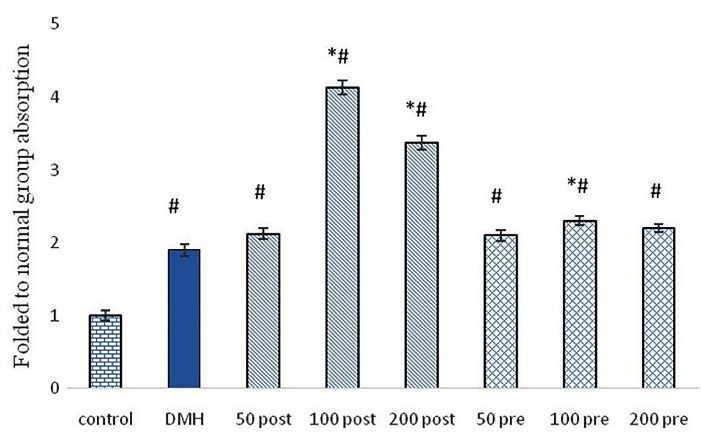
Effects of Scrophularia oxysephala (SO) extract on Caspase 3 in control and DMH (1,2-dimethylhydrazine) treated groups. Caspase 3 in control, DMH (1,2-dimethylhydrazine) and treatment groups received different doses of *Scrophularia oxysepala* (SO)methanolic extract. Animals received 50 mg/kg, 100 mg/kg and 200 mg/kg of extract after cancer induction called post-treatment groups (50 post, 100 post, and 200 post respectively) and animals which received 50 mg/kg, 100 mg/kg and 200 mg/kg of the extract before cancer induction called pre-treatment groups (50 pre, 100 pre, and 200 pre respectively). Data are presented as means (n = 8) ± SEM. * represent significant difference (*P* < 0.001) relative to DMH group. # represent significant difference (*P* < 0.001) relative to control group

The TUNEL method was used to investigate the apoptosis in the present study. Only cells that positively stained by TUNEL assay and revealed the typical morphological criteria of apoptosis, were counted as apoptotic. Apoptosis was seen in all groups. Pre-treatment and post-treatment with SO methanolic extract with different doses, significantly (*P* < 0.001) increased apoptosis compared to control and DMH groups. Post-treatment group treated with 100 mg/kg of SO extract showed the highest percent of apoptosis among treatment groups. Post-treatment groups showed significantly (*P*< < 0.01) higher percent of apoptosis compared with pre-treatment groups (Figures 4 and 5).

###  Caspase 3

**Figure 6:**
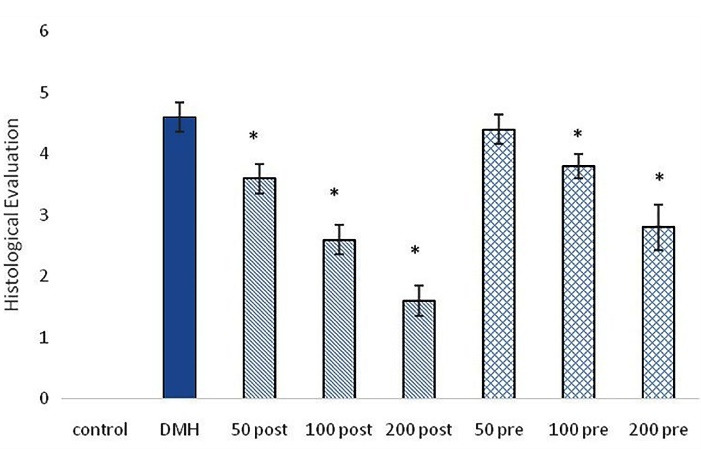
The effects of methanol extract of *Scrophularia oxysepala*(SO)on histology of DMH (1,2-dimethylhydrazine), 50 post, 100 post, 200 post (50 mg/kg, 100 mg/kg and 200 mg/kg, post-treatment respectively), 50 pre, 100 pre, 200 pre (50 mg/kg, 100 mg/kg and 200 mg/kg, pre-treatment respectively). Data are presented as means (n = 8) ± SEM. * represent significant difference (*P* < 0.001) relative to DMH groups

A specific ELISA technique was used to measure caspase 3 protein in the distal colon of control, DMH, 50 mg/kg, 100 mg/kg and 200 mg/kg, post-treatment and pre-treatment groups. Based on our results, pre-treatment and post-treatment significantly increased the fold of caspase 3 compared to the DMH group. The significantly higher increase in caspase three was observed in post-treatment groups compared to pre-treatment groups. Post-treatment groups treated with 100 mg/kg and 200 mg/kg showed the highest caspase 3 protein, which was three times higher than the control group and two times higher than the DMH group (Figure 6).

###  Histological evaluation

**Figure 7:**
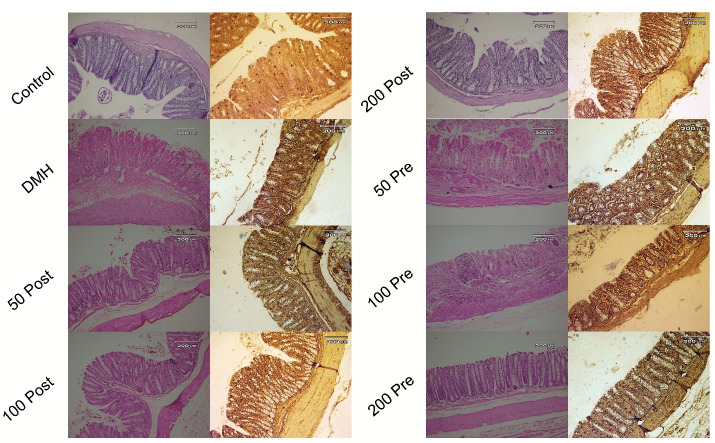
The effects of *Scrophularia oxysepala* (SO)methanolic extract on histological structure of DMH (1,2-dimethylhydrazine), 50 post, 100 post, 200 post (50 mg/kg, 100 mg/kg and 200 mg/kg, post-treatment respectively), 50 pre, 100 pre, 200 pre (50 mg/kg, 100 mg/kg and 200 mg/kg, pre-treatment respectively). H&E: Hematoxylin and eosin staining, TUNEL: Terminal deoxynucleotidyl transferase UTP nick end labeling method

Histological examination was performed on subjects treated with DMH, 50 mg/kg, 100 mg/kg and 200 mg/kg, post-treatment and pre-treatment groups, as described before. Pre-treatment and post-treatment with SO extract significantly improved histological epithelium structure compared to the DMH group. Intensive structural destruction was observed in DMH group. Destruction due to the DMH was improved in both pre-treatment and post-treatment groups. Best structural recovery results were observed in 100 mg/kg and 200 mg/kg post-treatment groups. Post-treatment groups significantly were more effective than pre-treatment groups (Figures 5 and 7).

Although tumorigenesis and carcinogenesis is a multistep procedure that initiated from a neoplastic cell.^26^ Experiments using chemical agents to induce pre-neoplastic lesions in animal models, which need less time to initiate carcinogenesis, provide a good endpoint to investigate and evaluate the effects of chemopreventive agents.^27^ In the present study, crypt foci, ACF, CM, apoptosis, caspase 3 protein, and histological evaluation was performed to determine the antineoplastic effects possible mechanisms of SO methanolic extract.

So far, the relation between ACF and colon cancer is not well understood; also, the correlation of ACF with colon cancer is discussed in some investigations.^15,28^ In this study, the number of AC, ACF, CM, caspase 3, apoptosis and histological evaluation predicts pre-treatment and post-treatment effects of SO extract on colon cancer. The SO extract effectively decreased the AC, ACF, and CM in all pre-treatment and post-treatment groups. Reducing the mentioned factors, especially ACF multiplicity was one of the interesting findings of the present study, especially in post-treatment groups. It is strongly accepted that large ACF can proceed to invasive cancer and malignancy than smaller ones.^15,29^ So, the ability of the extract to affect the CM especially in post-treatment and also pre-treatment groups is an interesting effect.

Pre-treatment was used to evaluate the preventive effects of SO extract in chemically induced colon cancer model and post-treatment, which is of more clinical importance since it can help to determine parameters helpful in recurrence and progression of precursor lesions. One of the mechanisms that may be involved in SO anticancer effects, especially in post-treatment groups are its strong anti-inflammatory properties, which can also impair synthesis of prostaglandins.^30^ Overexpression of cyclooxygenase-2 (COX-2) during inflammatory responses are believed to be linked to the different carcinogenesis steps that are colon cancer. Also, COX-2 is one of the mediators which control cell proliferation, and its inhibition with other inflammatory enzymes, inhibits cell proliferation, angiogenesis and activates apoptosis. Suppression of COX-2 also prevents the formation of DNA adducts. This might be in favor of promoting safe chemoprevention, although the majority of non-steroidal anti-inflammatory drugs cause GI ulcers due to COX-1 inhibition.

One of the most important cancer therapy depends on induction and increasing apoptosis to induce cell death in cancerous cells and the destruction of tumors.^31,32^ Consequently, to determine the possible effects of SO extract on activating and inducing apoptosis cascade, the caspase 3 as a critical protein in apoptosis and TUNEL assays were performed. Caspase 3 protein was evaluated using the ELISA method, and apoptosis was studied using the TUNEL method. A marked sign of the increase in caspase 3 and apoptosis was seen in pre-treatment and post-treatment groups compared with control and DMH groups.

TUNEL and caspace3 are used in literature for examining apoptosis in regards to natural products.^33,34^ The same results for caspase 3 protein were observed in Kilari et al study. In the mentioned study, the investigators evaluated the effects of pre-medication and post medication of *Basella rubra* aqueous extract (BRAE) in rats That received DMH. The results indicated that it uses significantly decreased the number of ACF. Histopathological findings showed a reduced number of goblet cells with a high level of dysplastic changes found only in DMH injected rats. However, in *Basella rubra* treated rats these changes were reversed. PCNA and Ki67 as cell proliferation expression markers were increased in DMH treated rats but reduced with BRAE treatment. These expressions were reversed for p53 and Caspase-3 as apoptosis markers. Accordingly, the obtained results indicate the potential efficacy of BRAE against colon carcinogenesis.^34^

Samanta et al studied the effect of micronutrient vanadium in inhibiting colon cancer induced by DMH. The results obtained from this report revealed that vanadium induces apoptosis in colon tumor, which confirmed by TUNEL assay. They proposed a positive correlation between the apoptotic index and p53 immunoexpression links connected to the vanadium-mediated apoptotic induction.^33^

## Conclusion

 In conclusion, the methanolic extract of SO showed chemopreventive potential by decreasing AC, ACF, and CM. Also, it could increase the caspase 3 protein and apoptosis in induced colon cancer cells. Anti-inflammatory and apoptosis induction are two main cancer chemopreventive mechanisms that might be involved in the activity of SO. Further studies are needed to determine whether these phytochemicals can induce apoptosis and inhibit the enzyme individually or in combination.

## Acknowledgments

 The authors would like to acknowledge Drug Applied Research Center at Tabriz University of Medical Sciences for the financial support.

## Ethical Issues

 Experiments were carried out in accordance with the guide for the Care and Use of Laboratory Animals and the protocol was approved by the Committee on Animal Research of Tabriz University of Medical Sciences.

## Conflict of Interest

 The authors declare that they have no conflict of interest.
